# Sodium Hyaluronate‐PDGF Repairs Cartilage and Subchondral Bone Microenvironment via HIF‐1α‐VEGF‐Notch and SDF‐1‐CXCR4 Inhibition in Osteoarthritis

**DOI:** 10.1111/jcmm.70515

**Published:** 2025-03-30

**Authors:** Zhengchao Wang, Pengfei Zhu, Hongmei Li, Bo Ye, Qiong Luo, Jiangxia Cheng, Yu Cai

**Affiliations:** ^1^ Department of Sports Medicine Wuhan Fourth Hospital Wuhan China; ^2^ Hubei Provincial Sports Medicine Center Wuhan China; ^3^ Department of Cardiovascular Wuhan Fourth Hospital Wuhan China; ^4^ Zibo First Hospital, Zibo Prevention and Treatment Hospital for Occupation Diseases Zibo China; ^5^ Department of Rehabilitation Wuhan Fourth Hospital Wuhan China; ^6^ Department of Anesthesiology Wuhan Fourth Hospital Wuhan China

**Keywords:** cartilage, microenvironment, osteoarthritis, platelet‐derived growth factor, subchondral bone

## Abstract

Chronic degenerative changes in cartilage and subchondral bone that lead to instability of the cartilage microenvironment are essential for the development of osteoarthritis (OA) in the old. Synchronous repair of cartilage and subchondral bone may be a key strategy for OA treatment. PDGF‐BB effectively promoted chondrocyte regeneration and angiogenesis. However, the mechanisms by which PDGF‐BB affects subchondral bone and the delivery of PDGF‐BB to the joint cavity need to be further explored. In this study, we used sodium hyaluronate to deliver PDGF‐BB (SH‐PDGF) to the joint space and aimed to determine the mechanisms of SH‐PDGF in repairing cartilage and subchondral bone and stabilising the cartilage microenvironment. In this research, we determined the pharmacokinetics of PDGF‐BB and SH‐PDGF in cartilage. Moreover, we investigated the effects of PDGF‐BB and SH‐PDGF on cartilage and the subchondral bone microenvironment by identifying changes in the HIF‐VEGF‐Notch axis and SDF‐1‐CXCR4 axis in an OA rat model. The results showed that PDGF‐BB increased cell viability, decreased HIF‐1α levels, inhibited inflammation and improved matrix metabolism in osteoarthritic chondrocytes under hyperoxic or hypoxic conditions. We also found that PDGF‐BB and SH‐PDGF showed similar effects on repairing cartilage and subchondral bone simultaneously. However, SH‐PDGF had some advantages over PDGF‐BB in prolonging the injection interval and decreasing the injection time. These protective effects were mediated by the inhibition of both the HIF‐1α‐VEGF‐Notch axis and the SDF‐1‐CXCR4 axis. The underlying mechanisms include the inhibition of HIF‐1α‐VEGF‐Notch‐mediated vessel invasion and SDF‐1‐CXCR4 axis‐mediated crosstalk between cartilage and subchondral tissue.

## 1 Introduction

The current clinical methods for treating osteoarthritis (OA) mainly relieve symptoms or delay disease progression, and few studies have focussed on repairing damaged cartilage and reversing the degenerative state of the joint. This issue is partly because the stability of the cartilage structure relies not only on the integrity of the cartilage layer but also on the support of the subchondral bone. Determining how to simultaneously improve the microenvironment between the cartilage layer and subchondral bone is crucial and important for repairing damaged cartilage in OA.

A previous study demonstrated that an appropriate level of hypoxia is essential for maintaining the microenvironmental stability of cartilage [1]. In contrast to cartilage, subchondral bone has abundant vessels, which can result in a hyperoxic microenvironment and increase the risk of an abnormal endochondral environment [2]. A barrier on the upper layer of subchondral bone and the lower layer of cartilage can isolate these two tissues to synchronously stabilise these microenvironments [3]. As OA progresses, this barrier can be destroyed by inflammation and abnormal stress, which promote vessel invasion from subchondral bone to cartilage [4]. Vessel invasion not only allows many cytokines to reach the cartilage but also causes the collapse of the hypoxic environment in the cartilage [4]. Under the combined influence of these effects, cartilage destruction is further aggravated, and OA becomes more severe [4].

Several mechanisms mediate the close connections between cartilage and subchondral bone. Previous studies revealed that the Smad signalling pathway is activated in cartilage during OA progression [5]. Moreover, Smad2/3 activation, which is involved in the RANKL signalling pathway, promotes osteoclast formation in subchondral bone [6]. Osteoclast formation further leads to the porosity of subchondral bone and causes abnormal signalling crosstalk between cartilage and subchondral bone. Coincidentally, SDF‐1 in the subchondral region can be easily transferred to cartilage and bind to CXCR4 in chondrocytes in OA [7]. Under these conditions, SDF‐1/CXCR4 regulates the ALK1/ALK5 ratio and in turn promotes cartilage damage via the Smad signalling pathway [7]. Furthermore, studies have shown that HIF‐1α widely exists in chondrocytes and can regulate morphogenesis and matrix synthesis in chondrocytes and inhibit chondrocyte apoptosis and autophagy [8, 9, 10]. In the initial stage of OA, inflammation‐ and hypoxia‐induced upregulation of HIF‐1α is a protective reaction against inflammatory damage [11, 12]. After the increased crosslinking of cartilage and subchondral bone, HIF‐1α overexpression activates the expression of VEGF and the Notch receptor [13]. The activated HIF‐1α‐VEGF‐Notch axis can mediate the elongation of local vessels and vessel invasion from subchondral bone to cartilage, leading to a change in the moderately hypoxic environment [14, 15, 16, 17]. Thus, HIF‐1α can exhibit bidirectional effects at different stages of OA development. Maintaining a relatively stable level of HIF‐1α in chondrocytes and cartilage is important for microenvironment stabilisation. Therefore, repairing the abnormal barrier and intervening in signal crosstalk between cartilage and subchondral bone are essential for preventing cartilage degeneration and OA progression.

Platelet‐derived growth factor (PDGF)‐BB derived from platelet‐rich plasma was first shown to promote vascular regeneration and subsequently was proven to have good potential in OA. Our previous study revealed that PDGF‐BB decreases caspase‐3‐dependent chondrocyte apoptosis [18]. In addition, PDGF‐BB can exhibit chondroprotective effects by decreasing the activity of the JAK2/STAT3 and p38/RunX‐2 signalling pathways, increasing the activity of the PKA/SOX‐9 signalling pathway and inhibiting the expression of CCAAT enhancer binding proteins δ and β [19]. As noted previously, PDGF‐BB can promote vascular regeneration, but whether this molecule can mediate chondroprotection by regulating the blood distribution in cartilage and subchondral bone is unclear. A previous study revealed that increasing the serum concentration of endogenous PDGF‐BB resulted in severe cartilage damage in OA via the promotion of angiogenesis in subchondral bone [20]. However, PDGF‐BB in serum may compensate for this increase; angiogenesis in subchondral bone is influenced by the local microenvironment, and the level of PDGF‐BB delivery from serum to subchondral bone cannot be accurately quantified. Our study also revealed that articular injection of exogenous PDGF‐BB promoted angiogenesis in subchondral bone, while almost no severe vascular infiltration was found in the cartilage layer [19]. In bone healing, PDGF‐BB binds to PDGFRβ to promote cell proliferation and angiogenesis in the early stage in response to bone injury, while this molecule can reduce Smad signalling and inhibit osteoclast differentiation in the late stage [21, 22]. Thus, PDGF‐BB affects the balance of Smad signalling during different disease processes. Therefore, PDGF‐BB may simultaneously repair cartilage and subchondral bone by affecting cartilage regeneration, angiogenesis and osteogenesis in OA.

As a small molecule drug, PDGF‐BB injected into the joint space can be easily absorbed and removed by capillaries and lymphatic vessels in the synovium [23]. This phenomenon limits our research on the use of PDGF‐BB for the treatment of chronic OA. Sodium hyaluronate (SH) can penetrate the injured tissue space and provide a scaffold for cell proliferation [24]. Moreover, its three‐dimensional structure can play a role in the sustained release of drugs [25]. In this study, we used SH to deliver PDGF‐BB in the joint space to decrease injection times and extend the duration of its effectiveness. Based on the slow release of this molecule, we aimed to explore the effects and underlying mechanisms of PDGF‐BB and SH‐PDGF in synchronously repairing cartilage and subchondral bone in OA.

## 2 Methods

### 2.1 Chondrocyte Isolation and Culture In Vitro

Seven‐day‐old neonatal male Sprague–Dawley rats were purchased from the Experimental Animal Center, Tongji Medical College, Huazhong University of Science and Technology. After aseptic exposure of the knee articular cavity, pieces of articular cartilage were dissected and separated from the underlying bone and connective tissues. The cartilage was then cut into 1 × 1 × 1 mm [3] pieces and washed three times with PBS. After 4–6 h of digestion with 1 g/L collagenase type II at 37°C, the suspension was centrifuged at 1000 rpm for 5 min to collect the chondrocytes. The extracted chondrocytes were cultured in DMEM/F‐12 supplemented with 10% fetal calf serum and 1% penicillin and streptomycin at a density of 1 × 10^5^ cells/mL (1 × 10^5^ cells/well) and incubated in a humidified atmosphere of 5% CO_2_ at 37°C. The culture medium was changed every 2–3 days, and the cells were passaged using a 0.25% trypsin–EDTA solution when 80%–90% confluence had been attained. Only the second or third generation of cells was used in the subsequent experiments. All experimental animals were maintained in accordance with the Guide for the Care and Use of Laboratory Animals of the National Institutes of Health, and the protocols were approved by the Ethics Committee of Wuhan Fourth Hospital (WAEF20240209).

### 2.2 Induction of Monosodium Iodoacetate (MIA)‐Induced Inflammation and PDGF‐BB Processing

Recombinant rat PDGF‐BB was purchased from R&D Systems, reconstituted and stored following the manufacturer's instructions. MIA was purchased from Aladdin, dissolved in normal saline and stored following the instructions. Chondrocytes were cultured in DMEM/F‐12 at a density of 1 × 10^5^ cells/mL in six‐well plates and incubated with PDGF‐BB (0 or 100 ng/mL) for 1 h prior to 5 μM MIA stimulation, as described in previous studies [18, 19]. Then, the cells were incubated with different oxygen concentrations (20%, 5%, 3%, 1%) for 24 h. The oxygen concentration in vitro was controlled by a tri‐gas incubator (Heracell 240i, Thermo Fisher, USA).

### 2.3 Cell Viability Assay and Enzyme‐Linked Immunosorbent Assays (ELISAs)

Cell viability was determined using a CCK‐8 kit according to the manufacturer's instructions. The absorbance at 450 nm was measured with a microplate reader (Leica Microsystems). ELISAs were conducted using commercial kits (R&D Systems) according to the manufacturer's instructions. All experiments were performed in triplicate.

### 2.4 Real‐Time Polymerase Chain Reaction (RT–PCR)

A total RNA kit (TaKaRa, Japan) was used to isolate RNA according to the manufacturer's instructions. An RNA PCR kit (TaKaRa) was used to conduct reverse transcription and cDNA synthesis. The RT‐PCR was performed using a Step One SYBR Green Mix Kit (TaKaRa) and an ABI Prism Sequence Detection System (Applied Biosystems, Waltham, MA) according to the manufacturer's instructions. The PCR primers used are shown in Table S1. The relative mRNA expression levels were calculated using the 2^ΔΔCT^ method.

### 2.5 Preparation of SH‐PDGF

Recombinant rat PDGF‐BB was purchased from R&D Systems and reconstituted in a 10 μg/mL solution. Then, the solution was mixed with a sufficient amount of SH (molecular weight 700,000–1,400,000) purchased from Bausch & Lomb Inc., to expand the volume by a factor of 100 and stir evenly. The concentration of PDGF‐BB in SH‐PDGF was also 100 ng/mL. The SH‐PDGF was injected within one hour after the preparation.

### 2.6 Animals

Male Sprague–Dawley (160–200 g) rats purchased from the Experimental Animal Center, Tongji Medical College, Huazhong University of Science and Technology, were fed a normal diet and housed under a 12‐h light/dark cycle under controlled humidity and temperature conditions (25°C). Left knee joint OA was induced by intraarticular injection of 3 mg/50 μL MIA under 1% pentobarbital anaesthesia [80 mg/kg intraperitoneally (i.p.)] using a 27‐gauge needle inserted through the patellar tendon. X‐ray analysis was performed after 2 weeks to evaluate changes in the joint structure using a Faxitron MX‐20 radiography system (Faxitron X‐ray Corp, Wheeling, IL). Radiographs were taken in the dorsoplantar position at full extension after euthanasia (1% pentobarbital anaesthesia, 80 mg/kg i.p.). The images were analysed by a radiologist who was blinded to the animal subgroups. Then, saline (control group), PDGF‐BB (100 ng/mL) and SH‐PDGF (100 ng/mL) were injected into the articular cavity of the left knee. After the last intervention, all animals (6 in each group, 30 in total) were killed using an overdose of 10% urethane. Pimonidazole hydrochloride (Hypoxyprobe) (60 mg/kg) was intraperitoneally injected one hour before the mice were sacrificed. The knee joints were dissected for histopathology. All experimental animals were maintained in accordance with the Guide for the Care and Use of Laboratory Animals of the National Institutes of Health, and the protocols were approved by the Ethics Committee of Wuhan Fourth Hospital. No animals died and were excluded during the experiments.

### 2.7 Histological Analysis

Rat knee joint samples were fixed in 4% paraformaldehyde for 24 h and then decalcified in 10% EDTA for 4 weeks. After paraffin embedding, 4‐μm‐thick sections were obtained for histological analysis. Haematoxylin and eosin (HE) staining and toluidine blue staining were carried out. The severity of the OA lesions was graded according to the Osteoarthritis Research Society International (OARSI) scoring system. En bloc basic fuchsin staining was conducted to measure microcracks as described in a previous study [26]. Microcracks were measured in 100‐μm‐thick sections of calcified cartilage (CC) and subchondral bone. Microcrack identification was based on the following criteria as described in a previous study: (1) intermediate in size, larger than canaliculi but smaller than vascular channels; (2) sharp borders with a halo of basic fuchsin staining; (3) stained through the depth of the section; and (4) edges of the cracks more deeply stained than the intervening space when the depth of focus was changed [26]. Microcracks were measured under light microscopy on one or two sections according to their quality. Three observers who were blinded to the grouping quantified the score and microcrack number, and three results were averaged.

### 2.8 Immunohistochemistry

After incubation at 60°C for 1 h, tissue sections were dewaxed in conventional xylene and rehydrated in gradient alcohol. The sections were incubated in 30% HO_2_ for 15 min to block endogenous peroxidase activity and then incubated in 10% normal sheep serum to block nonspecific binding. Then, the sections were incubated with an anti‐collagen II antibody (1:500, rabbit, CST) or anti‐collagen X antibody (1:300, rabbit, CST) overnight at 4°C. The sections were incubated with horseradish peroxidase (HRP)‐labelled goat anti‐rabbit IgG (1:500) secondary antibody at room temperature in the dark for 1 h. Colour development was performed with a DAB system. Immunostaining analysis was evaluated by measuring the ratio (%) of the positive area in the cartilage using the Optimas 6.5 software.

### 2.9 Fluorescence In Situ Hybridisation (FISH)

Slides were placed in a wet box, and 0.2 mol/L hydrochloric acid was applied to the tissue. We rinsed the slices for 15 min at room temperature and washed them twice with DEPC for 1 min each time. The slices were covered with proteinase K and placed in a molecular hybridiser at 37°C for 20 min. The proteinase K reaction was stopped with 0.2% or 0.1 mol/L glycine wash solution for 1 min, followed by two washes in PBS for 1 min each. We fixed the tissue with 4% paraformaldehyde (PFA) for 10 min and then washed it three times in PBS for 1 min each time. The slices were washed twice with acetic anhydride (pH = 8.0) at room temperature for 5 min, washed 5 times with PBS for 1 min each, and then washed twice with 5× SSC (pH 7.5) for 1 min each. The slices were placed in a wet box, and FISH probes were applied to the slides, which were then hybridised at 37°C overnight. Then, the slices were washed with 2 × SSC (pH = 7.5) at room temperature for 1 min, followed by washing five times with PBS for 1 min each at room temperature. Nuclei were stained with DAPI. The sequences of the probes are shown in Table S1.

### 2.10 Immunofluorescence (IF) Analysis

The histology slides were prepared as described earlier. The primary antibodies were diluted according to the manufacturer's instructions. After deparaffinisation and rehydration, the samples were incubated overnight with diluted primary antibodies at 4°C. Fluorescein‐labelled secondary antibodies were added to the samples, and the samples were incubated for 1 h at room temperature. Nuclei were stained with DAPI, and the slides were scanned using an LSM 710 confocal microscope (Zeiss, Oberkochen, Germany) with an EC‐Plan‐Neofluar 40×/1.3 oil immersion objective. Quantitative data were obtained from the fluorescence intensity measurements by using the ZEN 2009 software (Zeiss). The following primary antibodies were used: EMCN (1:500, rabbit, Proteintech), CD31 (1:500, rabbit, Proteintech), TRAP (1:200, rabbit, Proteintech), pimonidazole (1:50, mouse, Hypoxyprobe), HIF‐1α (1:500, rabbit, Proteintech), VEGF (1:300, rabbit, Proteintech), Notch1 (1:200, rabbit, Proteintech), Smad2 (1:500, rabbit, Proteintech), p‐Smad2 (1:200, rabbit, Proteintech), Smad3 (1:500, rabbit, Proteintech), p‐Smad3 (1:200, rabbit, Proteintech), NFATc1 (1:300, rabbit, Proteintech), SDF‐1 (1:300, rabbit, Proteintech), CXCR4 (1:200, rabbit, Proteintech), ALK1 (1:500, rabbit, Proteintech) and ALK5 (1:500, rabbit, Proteintech).

### 2.11 Statistical Analyses

Statistical analyses were performed using SPSS 21.0. The normality of the distribution was tested by a Q–Q plot. The data were analysed using repeated‐measures ANOVA. A post hoc test of the *p* value was performed with the Bonferroni correction. A value of *p* < 0.05 was considered statistically significant.

## 3 Results

### 3.1 Effects of PDGF‐BB on Cell Viability and HIF‐1α Expression Under Hyperoxic and Hypoxic Conditions In Vitro

MIA‐induced chondrocytes were incubated with 100 ng/mL PDGF‐BB at different oxygen concentrations (1%, 3%, 5%, 20%) for 24 h. Cell viability was measured by a CCK‐8 assay. As shown in Figure S1A, PDGF‐BB weakened the toxicity of MIA and increased cell viability. We next determined the levels of hypoxia‐related HIF‐1α by RT–PCR. Figure S1B shows that MIA‐induced inflammation increased HIF‐1α expression and that this effect was negatively correlated with the oxygen concentration. After PDGF‐BB treatment, the mRNA levels of HIF‐1α decreased significantly, while the HIF‐1α levels in the different oxygen concentration groups did not significantly differ. These results indicated that hypoxia further increased HIF‐1α expression without inhibiting cell viability in the MIA‐induced chondrocyte inflammation model. PDGF‐BB increased cell viability and decreased HIF‐1α levels under both hyperoxic and hypoxic conditions.

### 3.2 Effects of PDGF‐BB on Inflammation and Matrix Metabolism Under Hyperoxic and Hypoxic Conditions

The levels of inflammatory factors and metabolic markers in MIA‐induced chondrocytes treated with different oxygen concentrations (1%, 3%, 5%, 20%) with or without 100 ng/mL PDGF‐BB were measured via RT–PCR. Hypoxia had no apparent effect on the production of inflammatory factors (Figure S1C–E). PDGF‐BB decreased the mRNA levels of IL‐1, IL‐6 and TNF‐α under hyperoxic and hypoxic conditions. As shown in Figure S1F–I, MIA significantly increased catabolism (collagen X and MMP‐3) and weakened anabolism (collagen *II and aggrecan*), while these effects became weaker as the oxygen concentration decreased. Consistent with previous research, hypoxia induced the upregulation of HIF‐1α and improved matrix metabolism in MIA‐induced chondrocytes in the present study [27]. PDGF‐BB decreased the mRNA levels of collagen X and MMP‐3 and increased the mRNA levels of collagen II and aggrecan under hyperoxic and hypoxic conditions. These results demonstrated that PDGF‐BB inhibits inflammation and improves matrix metabolism in MIA‐induced chondrocytes under both hyperoxic and hypoxic conditions.

### 3.3 The Pharmacokinetics of PDGF‐BB and SH‐PDGF in Cartilage

**FIGURE 1:**
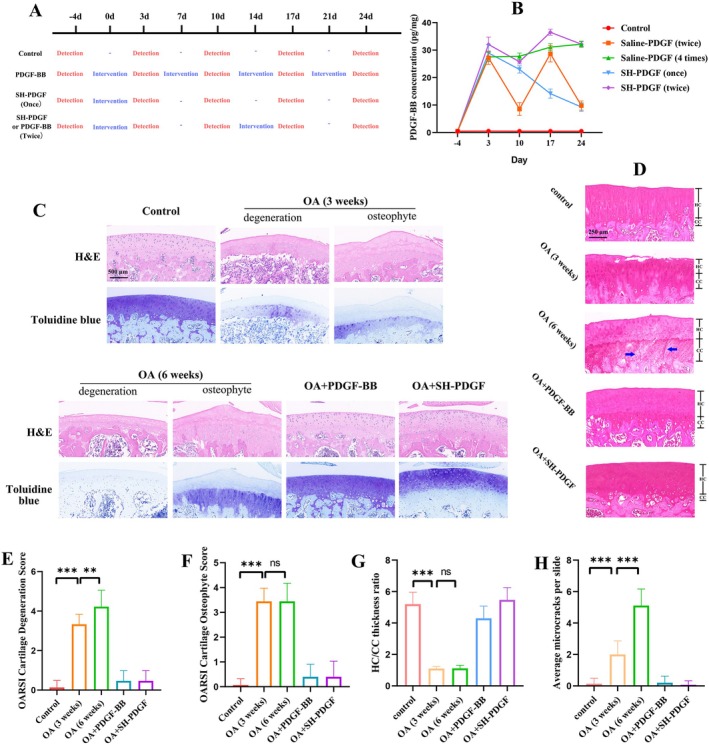
(A) The injection and detection strategy to obtain the time‐concentration plot of PDGF‐BB and SH‐PDGF. (B) Time‐concentration plot of PDGF‐BB and SH‐PDGF. Effects of PDGF‐BB and SH‐PDGF on histopathology in OA in vivo (5 rats in each group). (C) HE and toluidine blue staining of cartilage. (D) En bloc basic fuchsin staining of cartilage. (E and F) The OARSI score of cartilage. (G) The HC/CC thickness ratio in cartilage. (H) The number of microcracks in the CC and upper layer of subchondral bone. ns, no significant difference; **p < 0.01; ***p < 0.001.

To determine the proper injection times and intervals for SH‐PDGF treatment, we compared the concentration of PDGF‐BB in cartilage by ELISAs. As shown in Figure 1A, PDGF‐BB was injected every week for four weeks, as described in our previous studies, or every two weeks [18, 19]. SH‐PDGF was injected once every two weeks or only once every four weeks. Fifteen rats were included in each group, and three rats in each group were sacrificed every week to measure the PDGF‐BB concentration in the cartilage. The time–concentration plot is shown in Figure 1B. The PDGF‐BB concentration was similar between the PDGF‐BB group and the SH‐PDGF (twice) group, which indicates that prolonging the interval to two weeks of SH‐PDGF might have the same effect as PDGF‐BB injection per week. Based on the above results, we conducted joint cavity injections of PDGF‐BB per week or SH‐PDGF every two weeks for 4 weeks in subsequent experiments in vivo.

### 3.4 Histopathological Analysis of the Effects of PDGF‐BB and SH‐PDGF on Cartilage In Vivo

Cartilage degeneration and osteophyte formation were evaluated by HE and toluidine blue staining based on the OARSI scores (Figure 1C–F). Histological analysis revealed that MIA induced the loss of matrix in cartilage, which was accompanied by abnormal tissue hyperplasia and osteophyte formation in the impaired areas. The PDGF‐BB group and SH‐PDGF group presented significantly less matrix and chondrocyte loss than the MIA group and almost no cartilage tissue hyperplasia. The improvement in OARSI scores further verified the protective effect of PDGF‐BB on damaged cartilage. The microcracks in the CC and subchondral bone plate and the thickness ratio of the hyaline cartilage (HC)/CC were assessed by En bloc basic fuchsin staining. As shown in Figure 1G–I, PDGF‐BB and SH‐PDGF substantially decreased the occurrence of microcracks and increased the HC/CC thickness ratio. However, these effects did not significantly differ between the PDGF‐BB and SH‐PDGF groups. These results indicated that PDGF‐BB and SH‐PDGF exhibited similar protective effects in ameliorating cartilage damage, decreasing the porosity of the CC and subchondral bone, and maintaining structural stabilisation.

### 3.5 Effects of PDGF‐BB and SH‐PDGF on Inflammation and Matrix Metabolism in Cartilage In Vivo

Inflammation‐related factors in cartilage induced by MIA with or without PDGF‐BB and SH‐PDGF were measured by RT–PCR. The levels of matrix metabolic markers in cartilage were measured by RT–PCR and IF. As shown in Figure S1J, PDGF‐BB and SH‐PDGF did not significantly attenuate inflammation in osteoarthritic cartilage. As shown in Figure S1K–P, PDGF‐BB and SH‐PDGF both promoted catabolism and weakened anabolism in osteoarthritic cartilage, but the differences were not significant. These results were basically consistent with the in vitro results, while PDGF‐BB and SH‐PDGF exhibited the same effects on inflammation and matrix metabolism in MIA‐induced cartilage.

### 3.6 Effects of PDGF‐BB and SH‐PDGF on Osteoclast Formation and Angiogenesis in Subchondral Bone In Vivo

**FIGURE 2:**
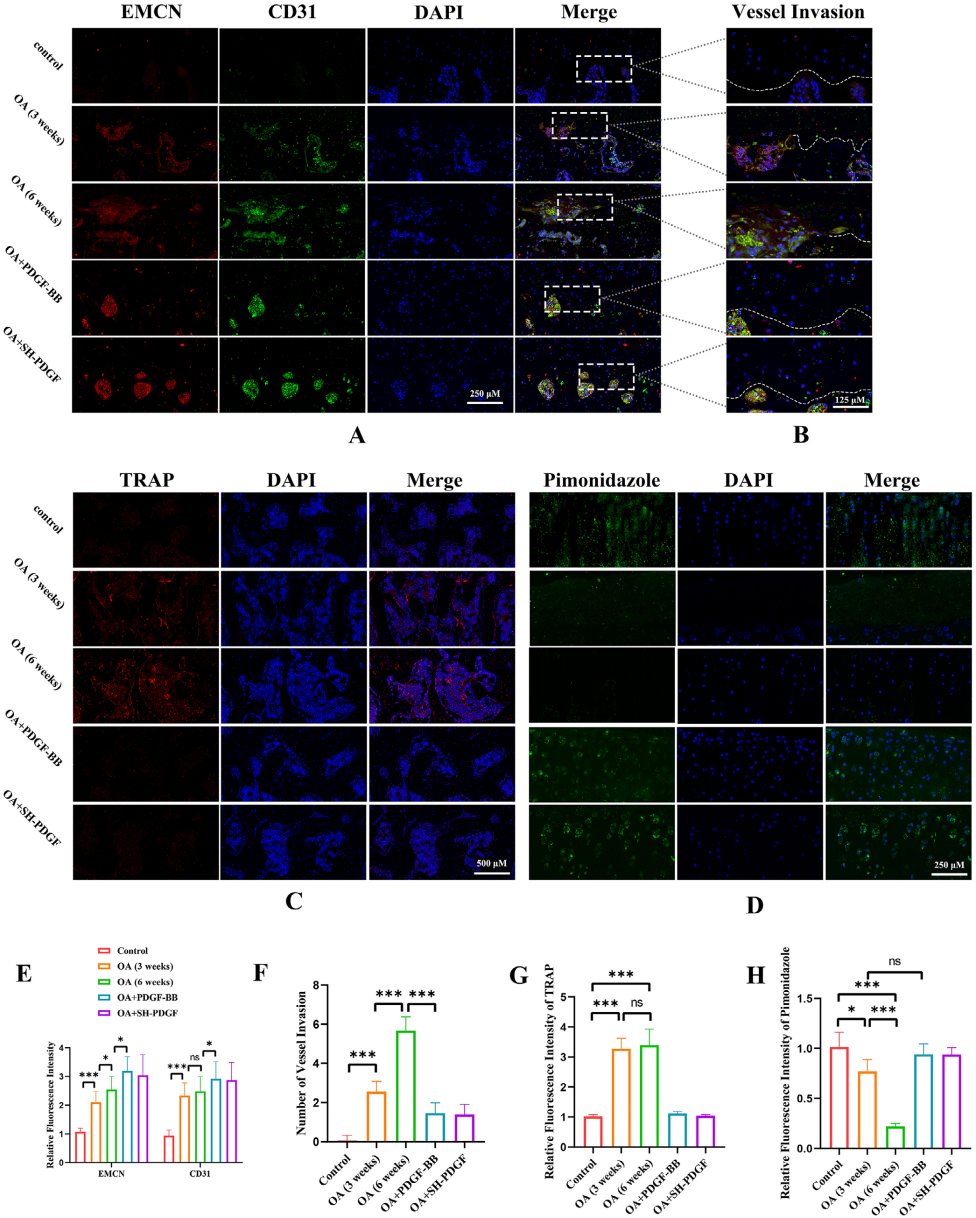
Effects of PDGF‐BB and SH‐PDGF on the microenvironment of cartilage and subchondral bone in OA in vivo (5 rats in each group). (A, B and E, F) Effects of PDGF‐BB and SH‐PDGF on angiogenesis and vessel invasion in subchondral bone. (C and G) Effects of PDGF‐BB and SH‐PDGF on osteoclast formation in subchondral bone determined by TRAP IF. (D and H) Effects of PDGF‐BB and SH‐PDGF on the hypoxic cartilage microenvironment, as shown by pimonidazole IF. ns, no significant difference; *p < 0.05; ***p < 0.001.

Osteoclast formation and angiogenesis in subchondral bone in vivo were analysed by CD31, EMCN, and TRAP IF. As shown in Figure 2C,G, PDGF‐BB and SH‐PDGF inhibited the increase in osteoclast formation in MIA‐induced cartilage and subsequently maintained the integrity of subchondral bone. Consistent with a previous study showing that PDGF‐BB increased angiogenesis, we also found that PDGF‐BB and SH‐PDGF further promoted subchondral bone angiogenesis after MIA induction (Figure 2A,E). To further elucidate the impact of angiogenesis in subchondral bone on cartilage, we then investigated vessel invasion, defined as the interruption of the border between cartilage and subchondral bone by vessels, at the junction of cartilage and subchondral bone. The results showed that vessel invasion was more severe at 6 weeks than at 3 weeks after MIA induction (Figure 2B,F). Vessel invasion from subchondral bone to cartilage was easily observed in the OA group but was barely detected in the PDGF‐BB and SH‐PDGF groups (Figure 2B,F). We concluded that PDGF‐BB and SH‐PDGF can attenuate subchondral bone destruction by inhibiting osteoclast formation and vessel invasion from subchondral bone to cartilage.

### 3.7 Effects of PDGF‐BB and SH‐PDGF on the Hypoxic Microenvironment and HIF‐1α Expression in Cartilage and Subchondral Bone In Vivo

As noted above, hypoxia in cartilage and hyperoxia in subchondral bone are essential for maintaining microenvironment stability, and damage to the barrier caused by increased microcracks and vessel invasion can alter this balance. Pimonidazole IF was used to evaluate the hypoxic environment in the cartilage, and the results showed that the fluorescence intensity of pimonidazole in the cartilage decreased significantly in the MIA‐induced cartilage at 6 weeks (Figure 2D,H). Moreover, more microcracks and vessel invasion were observed at 6 weeks than at 3 weeks. In contrast, PDGF‐BB and SH‐PDGF protected the barrier by inhibiting microcracks and vessel invasion and therefore maintaining the hypoxic microenvironment in cartilage.

**FIGURE 3:**
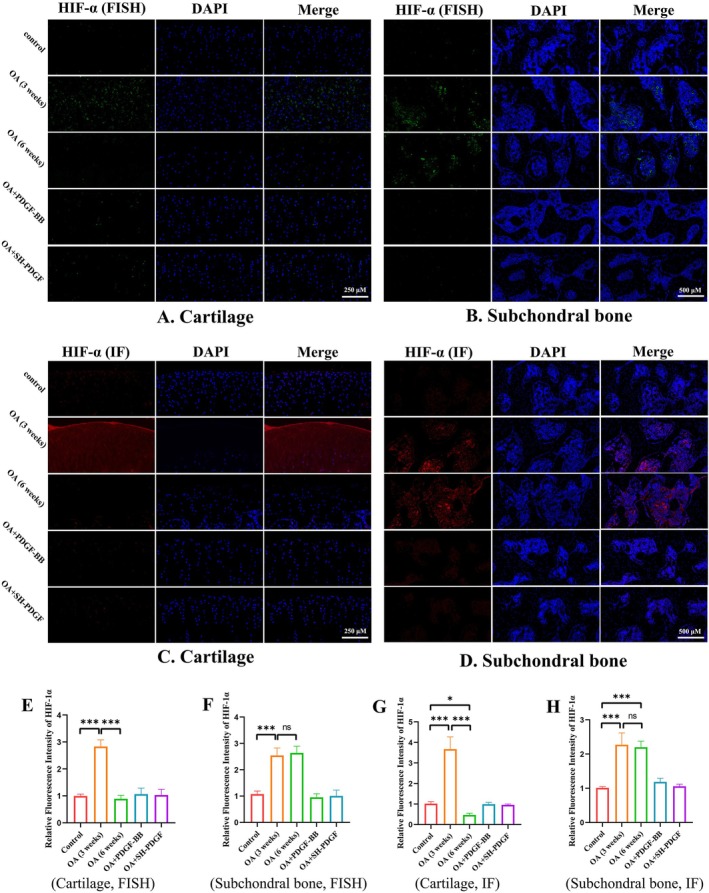
Effects of PDGF‐BB and SH‐PDGF on HIF‐1α expression in OA in vivo (5 rats in each group). (A and E) Effects of PDGF‐BB and SH‐PDGF on HIF‐1α expression in cartilage determined by FISH. (B and F) Effects of PDGF‐BB and SH‐PDGF on HIF‐1α expression in subchondral bone determined by FISH. (C and G) Effects of PDGF‐BB and SH‐PDGF on HIF‐1α expression in cartilage, as shown by IF. (D and H) Effects of PDGF‐BB and SH‐PDGF on HIF‐1α expression in subchondral bone determined by IF. ns, no significant difference; *p < 0.05; **p < 0.01; ***p < 0.001.

We next determined hypoxia‐related HIF‐1α expression in cartilage and subchondral bone by FISH and IF. In this study, HIF‐1α expression in cartilage exhibited two opposite changes at 3 and 6 weeks. As shown in Figure 3A,C,E,G, greater HIF‐1α expression and fewer microcracks were observed at 3 weeks, while less HIF‐1α expression and more microcracks were found at 6 weeks in the OA group. However, the HIF‐1α expression level in cartilage slightly improved after PDGF‐BB and SH‐PDGF injection. These effects in subchondral bone were different from those in cartilage. As shown in Figure 3B,D,F,H, MIA‐induced OA significantly upregulated HIF‐1α expression, while PDGF‐BB and SH‐PDGF downregulated HIF‐1α expression. We concluded that the ability of PDGF‐BB and SH‐PDGF to reverse the hypoxic microenvironment in cartilage might occur through multiple mechanisms, such as regulating HIF‐1α and decreasing microcracks and vessel invasion.

### 3.8 Effects of PDGF‐BB and SH‐PDGF on the HIF‐1α‐VEGF‐Notch Axis in Cartilage and Subchondral Bone In Vivo

**FIGURE 4:**
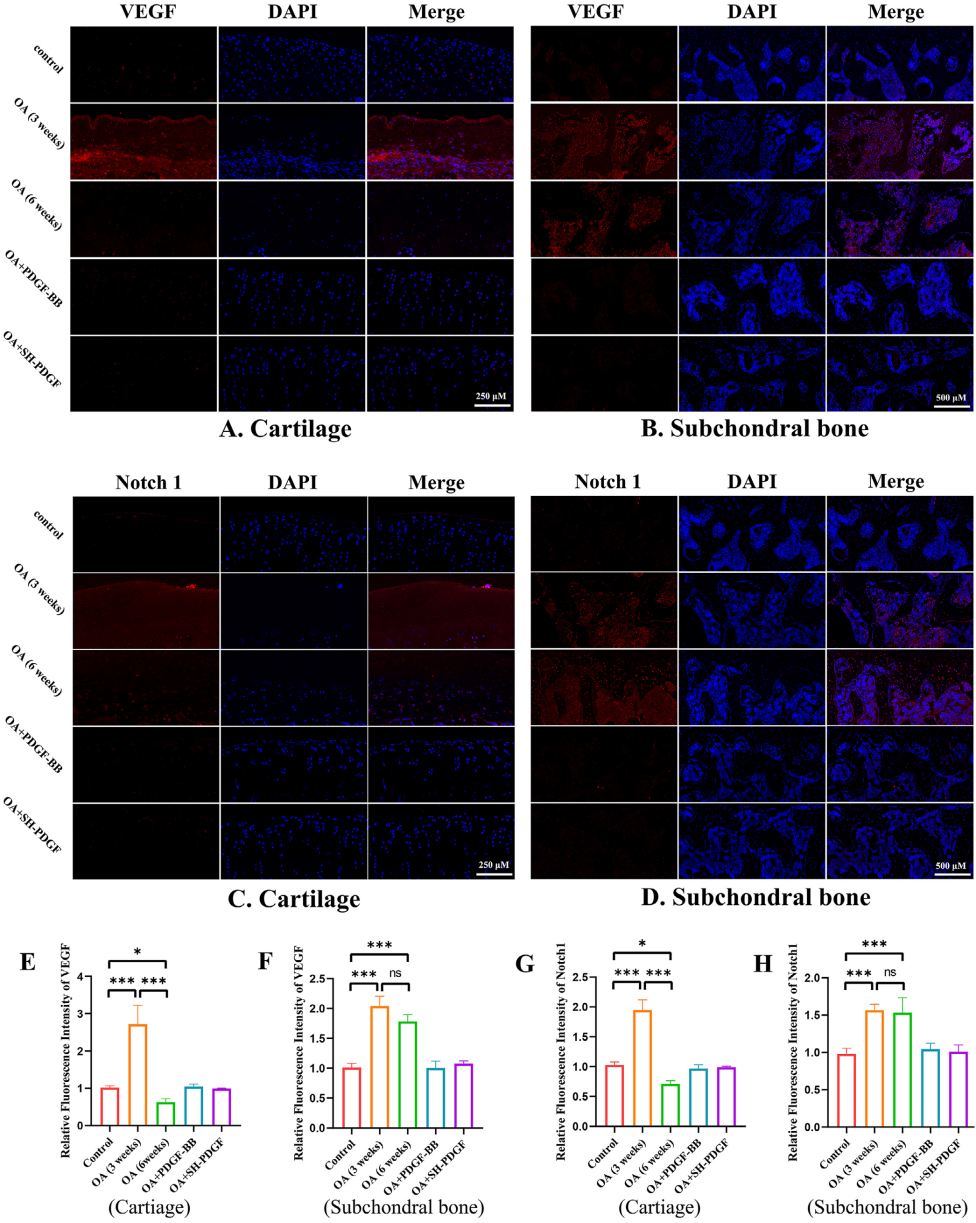
Effects of PDGF‐BB and SH‐PDGF on VEGF and Notch1 expression in OA in vivo (5 rats in each group). (A and E) Effects of PDGF‐BB and SH‐PDGF on VEGF expression in cartilage, as shown by IF. (B and F) Effects of PDGF‐BB and SH‐PDGF on VEGF expression in subchondral bone shown by IF. (C and G) Effects of PDGF‐BB and SH‐PDGF on Notch1 expression in cartilage, as shown by IF. (D and H) Effects of PDGF‐BB and SH‐PDGF on Notch1 expression in subchondral bone shown by IF. ns, no significant difference; *p < 0.05; **p < 0.01; ***p < 0.001.

The HIF‐1α‐VEGF‐Notch axis in cartilage and subchondral bone was assessed by IF. Similar to HIF‐1α expression, the VEGF and Notch1 levels tended to show different patterns in the early stage and late stage of MIA‐induced OA in cartilage. At 3 weeks, the fluorescence intensity of VEGF and Notch1 increased synchronously with that of HIF‐1α (Figure 4A,C,E,G). The results at 6 weeks showed that the expression levels of VEGF and Notch were both significantly decreased (Figure 4A,C,E,G). Treatment with PDGF‐BB and SH‐PDGF slightly reversed the inhibition of the HIF‐1α‐VEGF‐Notch axis in MIA‐induced OA. In subchondral bone, MIA‐induced OA significantly upregulated VEGF and Notch expression, while PDGF‐BB and SH‐PDGF decreased the levels of HIF‐1α, VEGF and Notch (Figure 4B,D,F,H). These results indicate that MIA‐induced inflammation promotes the HIF‐1α‐VEGF‐Notch axis in the early stage and that hyperoxia caused by the destruction of the hypoxic microenvironment can decrease the HIF‐1α‐VEGF‐Notch axis. Among these proteins, PDGF‐BB and SH‐PDGF can stabilise the HIF‐1α‐VEGF‐Notch axis both in cartilage and subchondral bone.

### 3.9 Effects of PDGF‐BB and SH‐PDGF on the SDF‐1‐CXCR4 Axis Between Cartilage and Subchondral Bone

In the progression of OA, the SDF‐1‐CXCR4 axis also participates in regulating cartilage and the subchondral microenvironment. We assessed the SDF‐1‐CXCR4 axis in cartilage and subchondral bone by FISH and IF.

**FIGURE 5:**
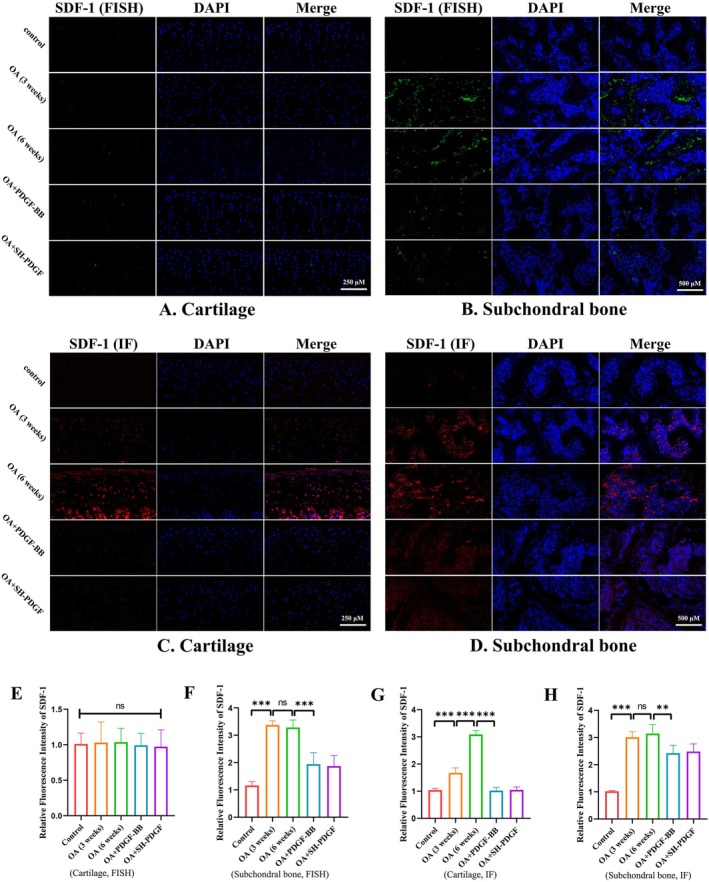
Effects of PDGF‐BB and SH‐PDGF on SDF‐1 expression in OA in vivo (5 rats in each group). (A and E) Effects of PDGF‐BB and SH‐PDGF on SDF‐1 expression in cartilage determined by FISH. (B and F) Effects of PDGF‐BB and SH‐PDGF on SDF‐1 expression in subchondral bone determined by FISH. (C and G) Effects of PDGF‐BB and SH‐PDGF on SDF‐1 expression in cartilage, as determined by IF. (D and H) Effects of PDGF‐BB and SH‐PDGF on SDF‐1 expression in subchondral bone determined by IF. ns, no significant difference; **p < 0.01; ***p < 0.001.

The FISH results showed that MIA‐induced OA with or without PDGF‐BB and SH‐PDGF did not affect the SDF‐1 RNA levels in cartilage (Figure 5A,E). However, the IF results shown in Figure 5C,G demonstrated that MIA‐induced OA upregulated SDF‐1 expression, which increased at 6 weeks. In addition, PDGF‐BB and SH‐PDGF decreased the SDF‐1 level in cartilage, which was close to that in the control group. Regarding SDF‐1 in subchondral bone, both PDGF‐BB and SH‐PDGF inhibited the upregulation of SDF‐1 in MIA‐induced OA (Figure 5B,D,F,H).

**FIGURE 6:**
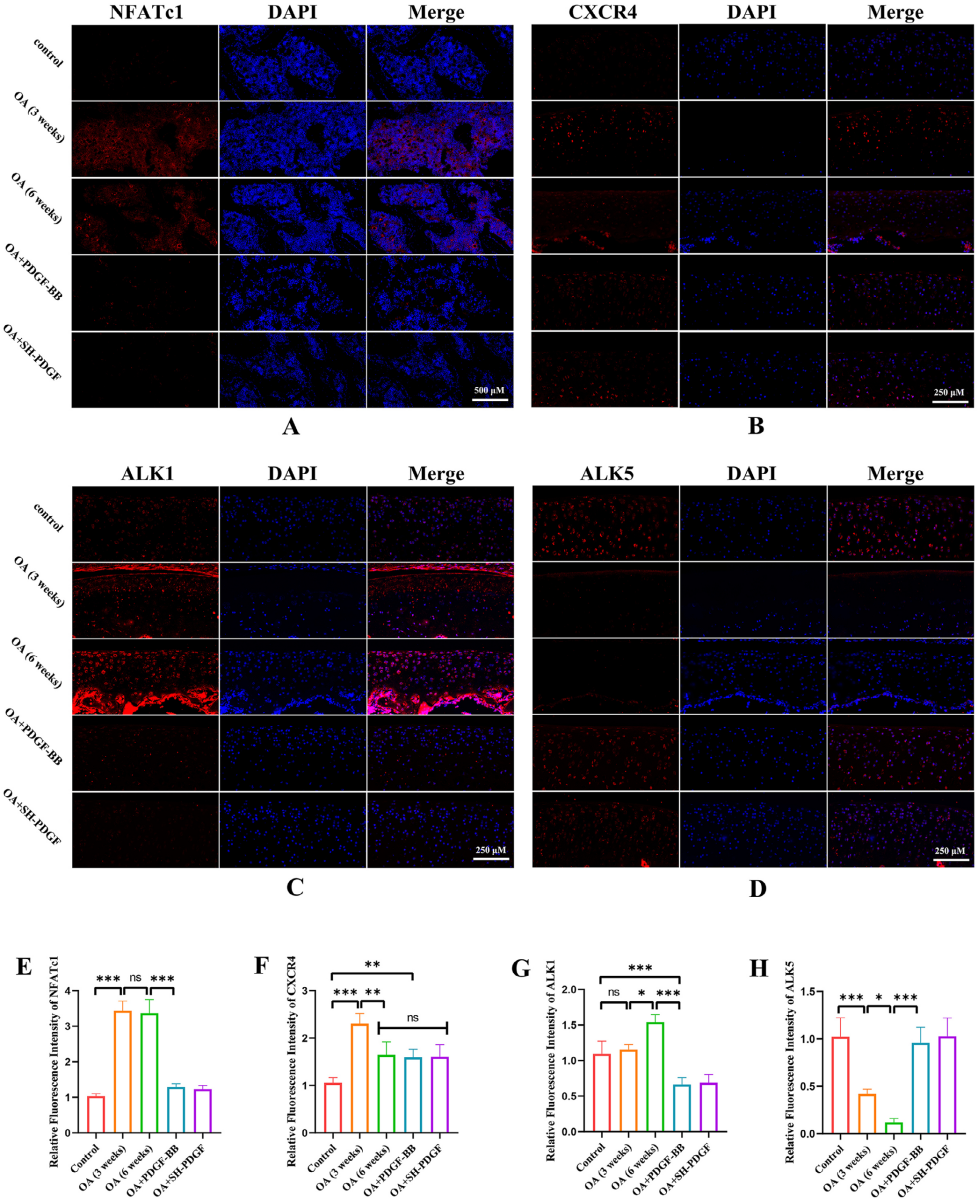
Effects of PDGF‐BB and SH‐PDGF on NFATc1, CXCR4, ALK1, and ALK5 expression in OA in vivo (5 rats in each group). (A and E) Effects of PDGF‐BB and SH‐PDGF on NFATc1 expression in subchondral bone determined by IF. (B and F) Effects of PDGF‐BB and SH‐PDGF on CXCR4 expression in cartilage, as shown by IF. (C and G) Effects of PDGF‐BB and SH‐PDGF on ALK1 expression in cartilage shown by IF. (D and H) Effects of PDGF‐BB and SH‐PDGF on ALK5 expression in cartilage, as shown by IF. ns, no significant difference; *p < 0.05; **p < 0.01; ***p < 0.001.

As shown in Figure 6B,F, MIA‐induced OA regulated the levels of CXCR4 in cartilage, and this effect was more obvious at 6 weeks than at 3 weeks. Interestingly, neither PDGF‐BB nor SH‐PDGF influenced the CXCR4 levels at 6 weeks (Figure 6B,F).

Although these results indicated that PDGF‐BB and SH‐PDGF have relatively weak direct inhibitory effects on SDF‐1 and CXCR4, these molecules decrease SDF‐1 transfer from subchondral bone to cartilage and inhibit SDF‐1‐CXCR4 axis‐mediated cartilage‐subchondral bone crosstalk.

### 3.10 Effects of PDGF‐BB and SH‐PDGF on ALK Expression and Smad2/3 Activation in Cartilage

The activin receptor‐like kinase 1/5 (ALK1/5) levels are associated with the metabolism of chondrocytes and mediate the phosphorylation of Smad2/3 [28]. The levels of ALK and Smad2/3 in cartilage were determined by IF. The ALK1 level in the OA group was similar to that in the control group at 3 weeks but tended to increase at 6 weeks (Figure 6C,G). In contrast to the change in ALK1, the ALK5 level at 6 weeks showed a more significant decrease than that at 3 weeks (Figure 6D,H). After PDGF‐BB or SH‐PDGF treatment, the ALK1 level decreased significantly and was lower than that in both the control and OA groups (Figure 6C,G). The ALK5 level was significantly increased and was close to that in the control group (Figure 6D,H). Smad2/3 activation has been shown to have antihypertrophic and anti‐inflammatory effects in cartilage [29]. The results showed that the phosphorylation of Smad2/3 was substantially increased at 3 weeks and was increased at 6 weeks after MIA‐induced OA (Figure S2). Compared with that in the OA group, the phosphorylation of Smad2/3 in the PDGF‐BB‐ or SH‐PDGF‐treated group was lower at 3 weeks but did not significantly change at 6 weeks.

These results indicated that both PDGF‐BB and SH‐PDGF significantly decreased the ALK1/ALK5 ratio in cartilage without obviously inhibiting Smad2/3 phosphorylation in cartilage.

### 3.11 Effects of PDGF‐BB and SH‐PDGF on Smad2/3 Activation and NFATc1 Expression in Subchondral Bone

A previous study demonstrated that p‐Smad2/3 activation in osteocytes in subchondral bone was positively associated with the severity of OA [6]. We next determined the phosphorylation level of Smad2/3 in subchondral bone by IF. As shown in Figure S3, the phosphorylation of Smad2/3 was significantly increased after 3 and 6 weeks of MIA induction. After PDGF‐BB or SH‐PDGF treatment, the Smad2/3 phosphorylation level significantly decreased in subchondral bone and was close to that in the control group. Moreover, NFATc1 is a key nuclear transcription factor that regulates osteoclast differentiation and subchondral osteosclerosis in OA [30]. NFATc1 expression in subchondral bone was also determined by IF. As shown in Figure 6A,E, the expression of NFATc1 was similar to that of phosphorylated Smad2/3, which was upregulated by OA induction and downregulated after PDGF‐BB and SH‐PDGF treatment. As described in a previous study, osteoclast formation depends on the activation of NFATc1, which is involved in the phosphorylation of Smad2/3 and the RANKL signalling pathway [6]. Therefore, our results indicated that PDGF‐BB and SH‐PDGF can inhibit osteoclast formation by inhibiting Smad2/3 phosphorylation and NFATc1 activation.

## 4 Discussion

Our study revealed the following findings: [1] PDGF‐BB can promote the simultaneous repair of cartilage and subchondral bone and can stabilise the cartilage microenvironment [2]. The inhibition of the HIF‐1α‐VEGF‐Notch axis and the SDF‐1‐CXCR4 axis in cartilage and subchondral bone might play important roles in this effect [3]. SH‐PDGF resulted in a similar effect to PDGF‐BB with prolonged injection intervals and decreased injection times.

### 4.1 PDGF‐BB Can Synchronously Repair Cartilage and Subchondral Bone in OA

Previous studies have demonstrated the close biochemical crosstalk between cartilage and subchondral bone during OA progression [31]. The HC and CC layers undergo various pathological changes, including degeneration, abnormal proliferation, and osteophyte formation, in response to stress, shearing, and inflammatory factors [26, 32]. Moreover, these pathological changes can cause abnormal incrassation and porosity in the CC layer [26]. Furthermore, abnormal changes in cartilage can be transmitted to subchondral bone by abnormal stress and activate the RANKL signalling pathway to promote osteoclast formation, causing porosity in subchondral bone [33]. The destruction of the CC and subchondral bone promotes vessel invasion and causes various cytokines to directly damage cartilage, which exacerbates OA [1].

Therefore, repair of only cartilage might be unsatisfactory, and synchronous subchondral bone repair is essential for OA treatment. We explored the cartilage repair and chondroprotective effects of PDGF‐BB in our previous studies [18, 19]. In this study, we investigated subchondral bone and found that PDGF‐BB inhibited osteoclast formation and reduced microcracks in the CC and subchondral bone. As a result, the simultaneous repair of cartilage and subchondral bone by PDGF‐BB could be an ideal intervention for OA.

### 4.2 PDGF‐BB Stabilises the Hypoxic Cartilage Microenvironment and Inhibits Vessel Invasion From Subchondral Bone to Cartilage by Inhibiting the HIF‐1α‐VEGF‐Notch Axis

HIF‐1α is an important transcription factor that can regulate metabolism, morphogenesis, and survival in chondrocytes [8, 9, 10]. However, this molecule is sensitive to the oxygen concentration in the microenvironment. Previous studies have shown that chondrocytes express more HIF‐1α under conditions of hypoxia or inflammation in vitro [8, 11]. However, another in vivo study revealed that HIF‐1α expression in an OA mouse model was lower than that in normal controls, possibly because of the collapse of the hypoxic environment caused by subchondral bone destruction and vessel invasion [1]. As noted above, barrier integrity is essential for maintaining a stable microenvironment in both cartilage and subchondral bone. Our results indicated that a relatively unbroken CC and subchondral bone barrier with less vessel invasion can maintain a stable hypoxic cartilage microenvironment and a high HIF‐1α level, while a relatively poor subchondral bone barrier with more vessel invasion can result in a hyperoxic cartilage microenvironment and a low HIF‐1α level. Although a recent study revealed that the hypoxic tolerance of chondrocytes might not be regulated by HIF‐1α, this molecule is still important for chondrocyte survival [34].

HIF‐1α levels change according to different stages of OA and regulate the cartilage microenvironment through different mechanisms. On the one hand, inflammation in OA can stimulate the expression of HIF‐1α, which can attenuate inflammatory damage and exhibit chondroprotective effects. On the other hand, a high HIF‐1α level can promote VEGF‐mediated angiogenesis and affect the hypoxic tissue microenvironment. Moreover, this change can induce the expression of the Notch receptor on the cell surface and in turn guide angiogenesis [14, 15]. This effect on the abnormal activity of the HIF‐1α‐VEGF‐Notch axis may lead to vessel invasion from subchondral bone to cartilage and promote the collapse of the cartilage microenvironment.

As a result, maintaining a proper HIF‐1α level that is not too high or too low may be essential for chondroprotection. We found that PDGF‐BB decreased HIF‐1α levels to a similar extent as those in the control group under both hyperoxia and hypoxia in vitro. Moreover, PDGF‐BB inhibited the HIF‐1α‐VEGF‐Notch axis in OA by preventing vessel invasion from subchondral bone to cartilage and stabilising the hypoxic environment of the cartilage. This finding can be explained by the downregulation of HIF‐1α by PDGF‐BB, which cooperates with its subchondral protective effects, preventing HIF‐1α in chondrocytes from being too high or too low and maintaining a hypoxic environment in cartilage. In addition, PDGF‐BB exhibited a powerful chondroprotective effect, as we previously found, and offset the decreasing chondroprotective effect of HIF‐1α [18, 19]. PDGF‐BB has been shown to activate the PI3K/Akt pathway, thereby enhancing HIF‐1α expression [35]; its effects may differ under inflammatory conditions. Previous studies have demonstrated that PI3K/Akt activation contributes to inflammation in osteoarthritis, while PDGF‐BB can inhibit PI3K/Akt activity under inflammatory induction [19, 36]. In our research, we observed that HIF‐1α was upregulated under inflammatory conditions, but the inhibitory effect of PDGF‐BB on HIF‐1α was not pronounced; it merely brought HIF‐1α levels closer to those of the control group under inflammation. Therefore, the observed opposite results may be attributed to the differential effects of PDGF‐BB under inflammatory conditions. Further studies are warranted to elucidate these mechanisms.

In our previous study, the promotion of angiogenesis in subchondral bone by PDGF‐BB and chondroprotection was self‐contradictory [19]. Here, we can explain this contradiction. First, this molecule can inhibit osteoclast formation, reduce microcracks in the CC and subchondral bone, and maintain the integrity of the barrier microenvironment. In addition, this treatment reduced the migration of vessels from subchondral bone to cartilage by regulating the HIF‐1α‐VEGF‐Notch axis. Angiogenesis in subchondral bone might not be related to OA severity, and vessel invasion is the critical point for OA progression. Therefore, further studies are needed to elucidate the complex crosstalk among the cell signalling pathway, angiogenesis, and tissue microenvironment.

### 4.3 PDGF‐BB Influences the Smad and SDF‐1‐CXCR4 Axes in Regulating Cartilage and the Subchondral Bone Microenvironment

The Smad pathway plays an important role in OA progression both in cartilage and in subchondral bone. The activation of Smad1/5/8 can contribute to the catabolism of the cartilage matrix [37]. The activation of Smad2/3 can contribute to the anabolism of the matrix and the formation of osteoclasts [6, 37]. Changes in the microenvironment of cartilage and subchondral bone can also affect Smad activation. On the one hand, HIF‐1α upregulation by hypoxia and inflammation can promote Smad activation [38, 39, 40]. On the other hand, the SDF‐1‐CXCR4 axis‐mediated cartilage‐subchondral crosstalk can disrupt the balance of ALK1/ALK5 in chondrocytes and in turn regulate the phosphorylation of Smad [7]. As the ALK1/ALK5 ratio increases, the catabolism mediated by Smad1/5/8 is increased, and the anabolism mediated by Smad2/3 is inhibited [41]. PDGF‐BB was shown to inhibit Smad activation [21, 22]. However, the effect of PDGF‐BB on Smad signalling may be more complex under the dynamic alteration of the microenvironment in cartilage and in subchondral bone. As shown above, Smad2/3 activation increased in both cartilage and subchondral bone at 3 weeks after MIA‐induced OA. Along with the destruction of the hypoxic microenvironment, Smad2/3 activation decreased in cartilage, followed by downregulation of HIF‐1α and activation of the SDF‐1‐CXCR4 axis. After PDGF‐BB or SH‐PDGF treatment, Smad2/3 activation in the subchondral region was significantly inhibited. In cartilage, PDGF‐BB inhibited Smad2/3 activation while simultaneously reducing the effect of inhibition of the SDF‐1‐CXCR4 axis on the ALK1/ALK5 ratio and promoting Smad2/3 activation. Under these combined conditions, the level of Smad2/3 activation did not significantly differ between the OA group and the PDGF‐BB group at 6 weeks. These findings indicated that the changes in the microenvironment of cartilage and subchondral bone are also attributed to the regulation of the Smad signalling pathway, and it is inappropriate to explain such effects through a single signalling pathway.

In addition, PDGF‐BB can regulate the SDF‐1‐CXCR4 axis and influence cartilage and the subchondral microenvironment. CXCR4 in cartilage and SDF‐1 in subchondral bone were significantly upregulated under inflammatory induction [7]. Along with the destruction of the subchondral barrier, SDF‐1 in subchondral bone can be transferred to cartilage and bind to CXCR4 in chondrocytes [7]. Another study showed that downregulation of HIF‐1α decreased CXCR4 expression [42]. In this study, our results were basically consistent with those of previous studies. After 3 weeks of MIA‐induced OA, SDF‐1 in cartilage and subchondral bone and CXCR4 in cartilage were significantly upregulated. After the destruction of the subchondral barrier at 6 weeks, SDF‐1 was transferred from the subchondral bone to the cartilage. However, CXCR4 expression decreased in an opposite pattern at 6 weeks due to the destruction of the hypoxic cartilage microenvironment and downregulation of HIF‐1α. After PDGF‐BB or SH‐PDGF treatment, CXCR4 in cartilage and SDF‐1 in subchondral bone changed only slightly compared to those in the OA group at 6 weeks. However, the SDF‐1 level in cartilage dramatically decreased, which indicated that the SDF‐1‐CXCR4 axis was inhibited by PDGF‐BB. We concluded that PDGF‐BB does not have a direct effect on SDF‐1 or CXCR4. However, the repair effect of the subchondral bone barrier can inhibit the transformation of subchondral SDF‐1 to cartilage, which indirectly inhibits the SDF‐1‐CXCR4 axis. These findings also indicate that the simultaneous repair of subchondral bone and cartilage and cartilage microenvironment stabilisation are important.

### 4.4 Compared With PDGF‐BB, SH‐PDGH‐BB has Good Potential for Repairing Subchondral Bone and Cartilage Simultaneously

Clinical studies have shown that platelet‐rich plasma (PRP) can benefit patients suffering from musculoskeletal disorders, including OA [43, 44]. However, the major limitations of this treatment include high demand, repeated administration, local immune inflammation, and haematological abnormalities. More importantly, not all cytokines in PRP, such as VEGF, TNF‐α and SDF‐1, which could be harmful to cartilage and promote OA progression, benefit OA patients [45]. PDGF‐BB is one of the most important cytokines in PRP, and injections in the joint space can be easily absorbed and eliminated by capillaries and lymphatic vessels in the synovium [23]. SH is also widely applied in OA treatment because it fills the gap between lesions and acts as a scaffold for cell proliferation [24, 25, 46]. Given the advantages of SH in treating OA, we used SH to dissolve PDGF‐BB to act as a carrier for PDGF‐BB injection and to prolong the interval and reduce intervention times. Our results showed that SH‐PDGF partially compensated for the pharmacokinetic defects of PDGF‐BB and thus reduced the number of injections of PDGF‐BB, resulting in the same effects. We found that PDGF‐BB combined with SH can synergistically repair the cartilage and subchondral bone microenvironment.

**FIGURE 7:**
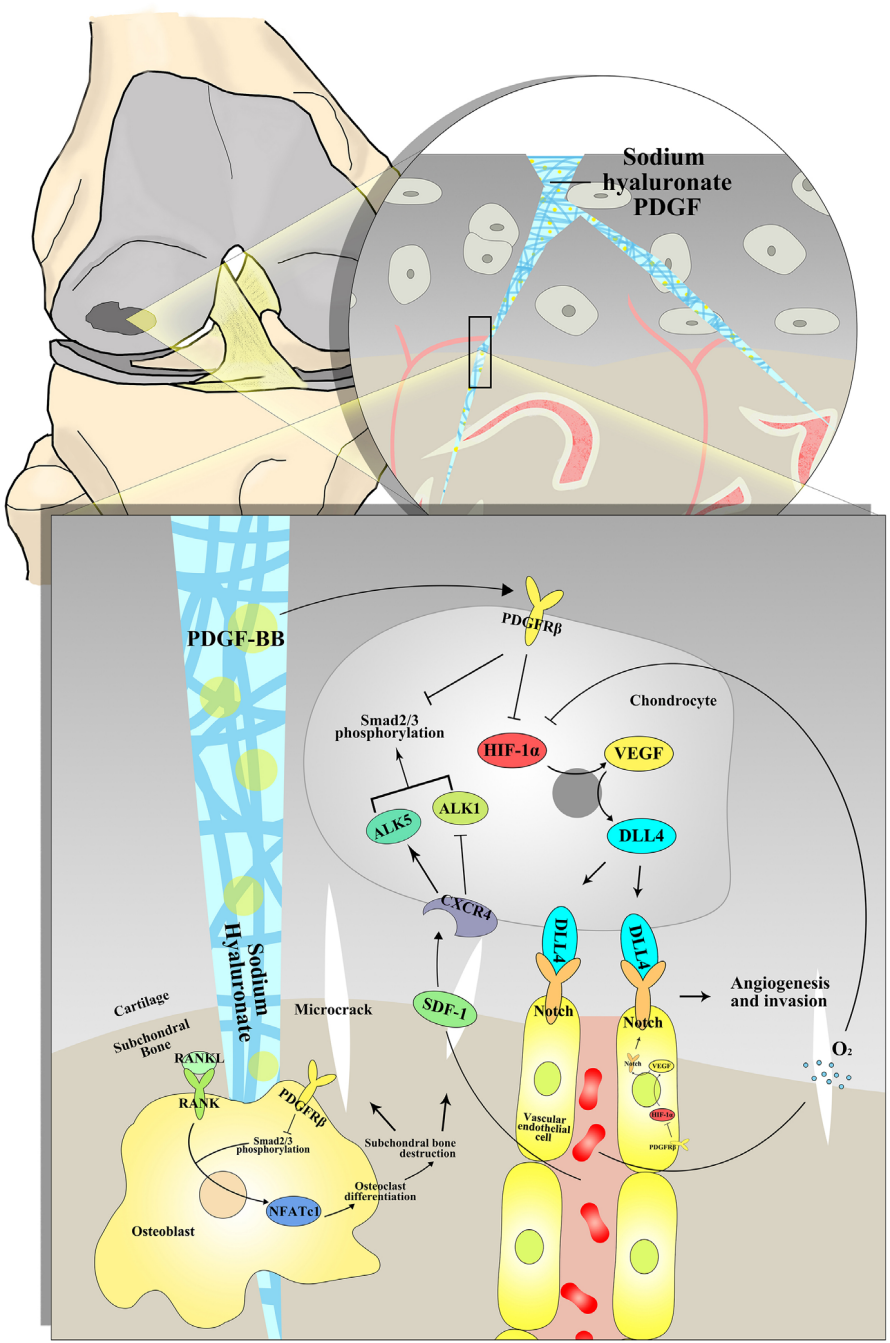
Schematic model of the effects and mechanisms of PDGF‐BB and SH‐PDGF on synchronously repairing cartilage and subchondral bone and stabilising the cartilage microenvironment in OA.

In conclusion, as shown in Figure 7, this study demonstrated that PDGF‐BB alleviated OA by simultaneously repairing cartilage and subchondral bone and stabilising the cartilage microenvironment. The underlying mechanisms include the inhibition of HIF‐1α‐VEGF‐Notch‐mediated vessel invasion and SDF‐1‐CXCR4 axis‐mediated crosstalk between cartilage and subchondral bone. Our findings also indicate that synchronous repair of cartilage and subchondral bone may be a key strategy for OA treatment. In addition, SH can be applied as a carrier for intra‐articular injection of PDGF‐BB to decrease the injection time and prolong the injection interval without weakening the therapeutic effect.

## Author Contributions

**Zhengchao Wang:** conceptualization (equal), data curation (equal), formal analysis (equal), funding acquisition (equal), project administration (equal), software (equal), supervision (equal), writing – original draft (equal). **Pengfei Zhu:** conceptualization (equal), data curation (equal), project administration (equal), software (equal), validation (equal), writing – original draft (equal). **Hongmei Li:** conceptualization (equal), investigation (equal), methodology (equal), project administration (equal), writing – original draft (equal). **Bo Ye:** investigation (equal), methodology (equal). **Qiong Luo:** investigation (equal), methodology (equal). **Jiangxia Cheng:** conceptualization (equal), data curation (equal), formal analysis (equal), project administration (equal), software (equal), supervision (equal), writing – review and editing (equal). **Yu Cai:** conceptualization (equal), data curation (equal), investigation (equal), methodology (equal), project administration (equal), validation (equal), writing – original draft (equal), writing – review and editing (equal).

## Ethics Statement

All experimental animals were maintained in accordance with the Guide for the Care and Use of Laboratory Animals of the National Institutes of Health and were approved by the Ethics Committee for Animal Experimentation of Wuhan Fourth Hospital (No. WAEF20240209).

## Conflicts of Interest

The authors declare no conflicts of interest.

## Supporting information


**Figure S1.** (A–I) Effects of hypoxia and PDGF‐BB on osteoarthritic chondrocytes in vitro. (A) Effects of hypoxia and PDGF‐BB on cell viability. (B) Effects of hypoxia and PDGF‐BB on HIF‐1α expression. (C–E) Effects of hypoxia and PDGF‐BB on inflammatory factors. (F–I) Effects of hypoxia and PDGF‐BB on the expression of matrix metabolic markers. *p < 0.05 vs. the control group; ***p < 0.001 vs. the control group; ^###^ *p* < 0.001 vs. the 20% O_2_ + MIA group. (J‐P) Effects of PDGF‐BB and SH‐PDGF on inflammation and matrix metabolism in OA in vivo (5 rats in each group). (J) Effects of PDGF‐BB and SH‐PDGF on inflammatory factors identified by RT–PCR. (K‐L) Effects of PDGF‐BB and SH‐PDGF on matrix metabolic markers identified by RT–PCR. (M‐P) Effects of PDGF‐BB and SH‐PDGF on the expression of matrix metabolic markers identified by immunohistochemistry. ns, no significant difference; *p < 0.05; **p < 0.01; ***p < 0.001.

**Figure S2.** Effects of PDGF‐BB and SH‐PDGF on Smad2/3 phosphorylation in osteoarthritic cartilage in vivo (5 rats in each group). (A, B and E, F) Effects of PDGF‐BB and SH‐PDGF on Smad2 phosphorylation in cartilage, as shown by IF. (C, D and G, H) Effects of PDGF‐BB and SH‐PDGF on Smad3 phosphorylation in cartilage, as shown by IF. ns, no significant difference; *p < 0.05; **p < 0.01; ***p < 0.001.

**Figure S3.** Effects of PDGF‐BB and SH‐PDGF on Smad2/3 phosphorylation in osteoarthritic subchondral bone in vivo. (A, B and E, F) Effects of PDGF‐BB and SH‐PDGF on Smad2 phosphorylation in subchondral bone, as shown by IF. (C, D and G, H) Effects of PDGF‐BB and SH‐PDGF on Smad3 phosphorylation in subchondral bone, as shown by IF. ns, no significant difference; *p < 0.05; **p < 0.01; ***p < 0.001.

**Table S1.** Sequence for PCR primers and FISH probes.

## Data Availability

The datasets used and/or analysed during this study are available from the corresponding author upon reasonable request.
