# Supplementary figures and images for: Sodium Hyaluronate‐PDGF Repairs Cartilage and Subchondral Bone Microenvironment via HIF‐1α‐VEGF‐Notch and SDF‐1‐CXCR4 Inhibition in Osteoarthritis
Source: J Cell Mol Med. 2025 Mar 30;29(7):e70515. doi: 10.1111/jcmm.70515 (PMC11955409; doi:10.1111/jcmm.70515)

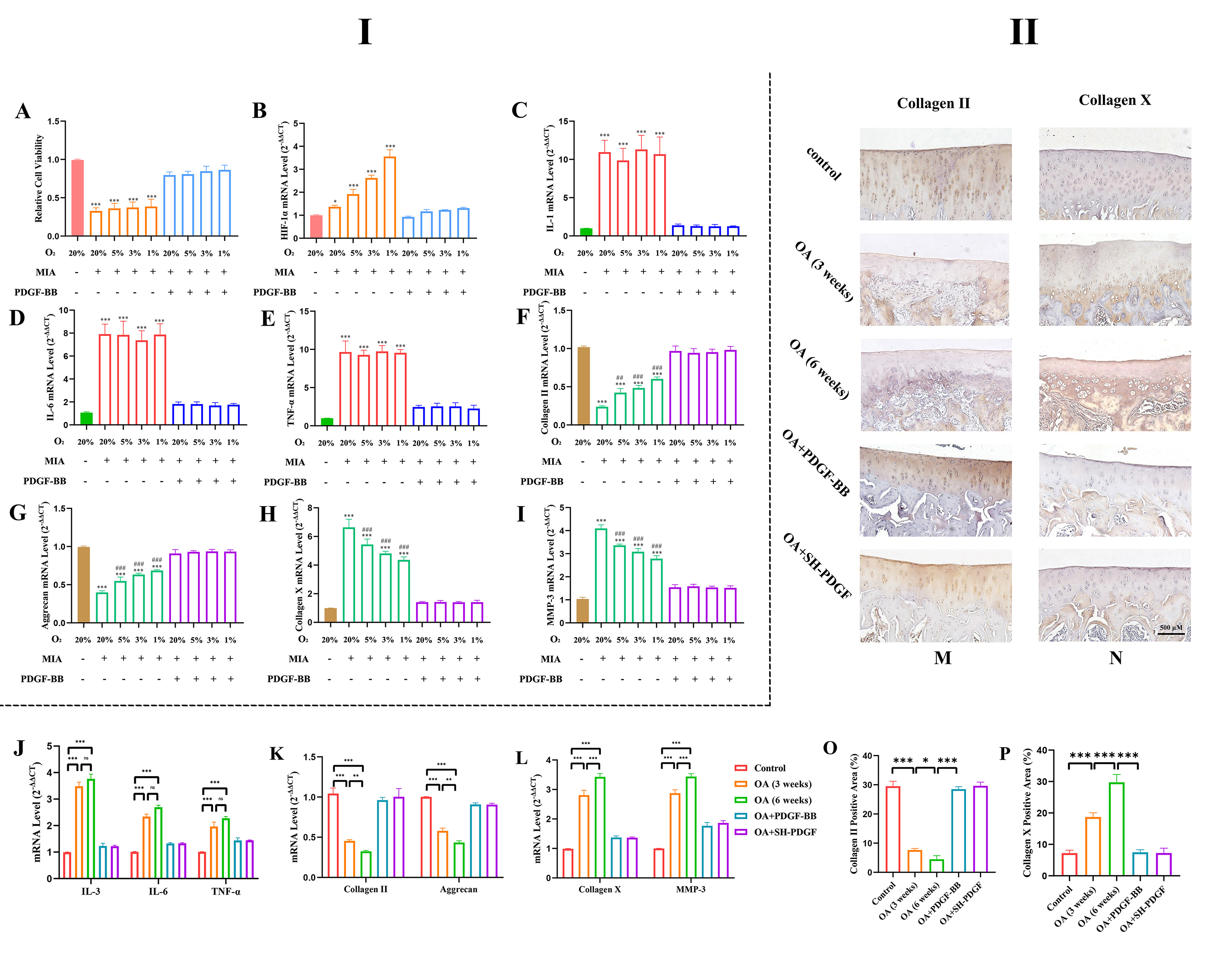

Supplement: Supplementary file 1 — Figure S1. (A–I) Effects of hypoxia and PDGF‐BB on osteoarthritic chondrocytes in vitro. (A) Effects of hypoxia and PDGF‐BB on cell viability. (B) Effects of hypoxia and PDGF‐BB on HIF‐1α expression. (C–E) Effects of hypoxia and PDGF‐BB on inflammatory factors. (F–I) Effects of hypoxia and PDGF‐BB on the expression of matrix metabolic markers. *p < 0.05 vs. the control group; ***p < 0.001 vs. the control group; ### p < 0.001 vs. the 20% O2 + MIA group. (J‐P) Effects of PDGF‐BB and SH‐PDGF on inflammation and matrix metabolism in OA in vivo (5 rats in each group). (J) Effects of PDGF‐BB and SH‐PDGF on inflammatory factors identified by RT–PCR. (K‐L) Effects of PDGF‐BB and SH‐PDGF on matrix metabolic markers identified by RT–PCR. (M‐P) Effects of PDGF‐BB and SH‐PDGF on the expression of matrix metabolic markers identified by immunohistochemistry. ns, no significant difference; *p < 0.05; **p < 0.01; ***p < 0.001. [file JCMM-29-e70515-s002.jpg]

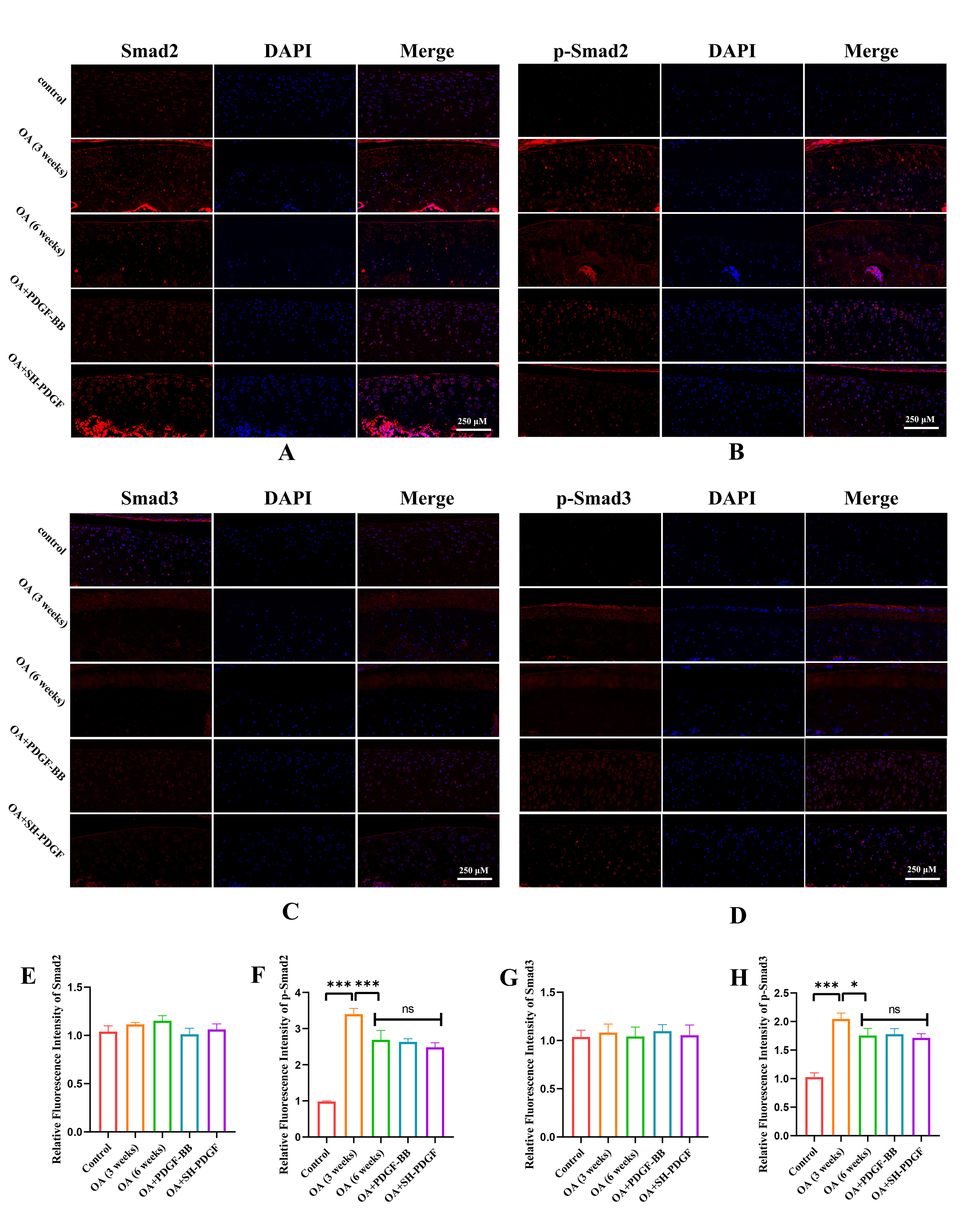

Supplement: Supplementary file 2 — Figure S2. Effects of PDGF‐BB and SH‐PDGF on Smad2/3 phosphorylation in osteoarthritic cartilage in vivo (5 rats in each group). (A, B and E, F) Effects of PDGF‐BB and SH‐PDGF on Smad2 phosphorylation in cartilage, as shown by IF. (C, D and G, H) Effects of PDGF‐BB and SH‐PDGF on Smad3 phosphorylation in cartilage, as shown by IF. ns, no significant difference; *p < 0.05; **p < 0.01; ***p < 0.001. [file JCMM-29-e70515-s004.jpg]

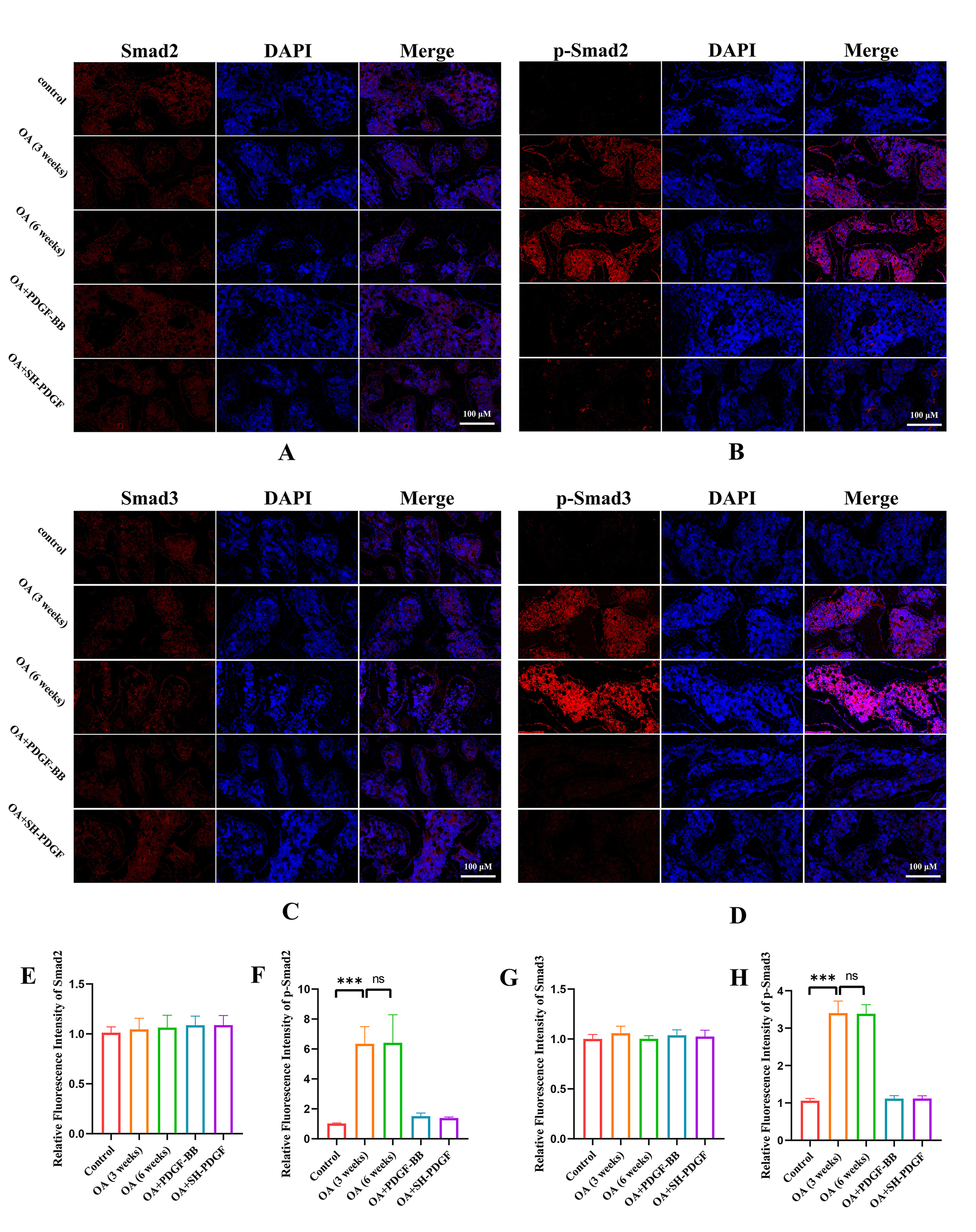

Supplement: Supplementary file 3 — Figure S3. Effects of PDGF‐BB and SH‐PDGF on Smad2/3 phosphorylation in osteoarthritic subchondral bone in vivo. (A, B and E, F) Effects of PDGF‐BB and SH‐PDGF on Smad2 phosphorylation in subchondral bone, as shown by IF. (C, D and G, H) Effects of PDGF‐BB and SH‐PDGF on Smad3 phosphorylation in subchondral bone, as shown by IF. ns, no significant difference; *p < 0.05; **p < 0.01; ***p < 0.001. [file JCMM-29-e70515-s003.jpg]
