# Supplementary material for: Sodium Hyaluronate‐PDGF Repairs Cartilage and Subchondral Bone Microenvironment via HIF‐1α‐VEGF‐Notch and SDF‐1‐CXCR4 Inhibition in Osteoarthritis
Source: J Cell Mol Med. 2025 Mar 30;29(7):e70515. doi: 10.1111/jcmm.70515 (PMC11955409; doi:10.1111/jcmm.70515)
Supplement: Supplementary file 4 — Table S1. Sequence for PCR primers and FISH probes. [file JCMM-29-e70515-s001.docx]

**Figure S1.** (A-I) Effects of hypoxia and PDGF-BB on osteoarthritic chondrocytes in vitro. (A) Effects of hypoxia and PDGF-BB on cell viability. (B) Effects of hypoxia and PDGF-BB on HIF-1α expression. (C-E) Effects of hypoxia and PDGF-BB on inflammatory factors. (F-I) Effects of hypoxia and PDGF-BB on the expression of matrix metabolic markers. *, *p* < 0.05 vs. the control group; ***, *p* < 0.001 vs. the control group; ###, *p* < 0.001 vs. the 20% O2+MIA group.

(J-P) Effects of PDGF-BB and SH-PDGF on inflammation and matrix metabolism in OA in vivo (5 rats in each group). (J) Effects of PDGF-BB and SH-PDGF on inflammatory factors identified by RT‒PCR. (K-L) Effects of PDGF-BB and SH-PDGF on matrix metabolic markers identified by RT‒PCR. (M-P) Effects of PDGF-BB and SH-PDGF on the expression of matrix metabolic markers identified by immunohistochemistry. ns, no significant difference; *, *p* < 0.05; **, *p* < 0.01; ***, *p* < 0.001.

**Figure S2.** Effects of PDGF-BB and SH-PDGF on Smad2/3 phosphorylation in osteoarthritic cartilage in vivo (5 rats in each group). (A-B and E-F) Effects of PDGF-BB and SH-PDGF on Smad2 phosphorylation in cartilage, as shown by IF. (C-D and G-H) Effects of PDGF-BB and SH-PDGF on Smad3 phosphorylation in cartilage, as shown by IF. ns, no significant difference; *, *p* < 0.05; **, *p* < 0.01; ***, *p* < 0.001.

**Figure S3.** Effects of PDGF-BB and SH-PDGF on Smad2/3 phosphorylation in osteoarthritic subchondral bone in vivo. (A-B and E-F) Effects of PDGF-BB and SH-PDGF on Smad2 phosphorylation in subchondral bone, as shown by IF. (C-D and G-H) Effects of PDGF-BB and SH-PDGF on Smad3 phosphorylation in subchondral bone, as shown by IF. ns, no significant difference; *, *p* < 0.05; **, *p* < 0.01; ***, *p* < 0.001.

**Table S1. Sequence for PCR primers and FISH probes**

| Gene | Sequence (5’ -3’) |
| --- | --- |
| HIF-1α | F: 5’-TCACTGTACATGCCACCGCAA-3’ |
|  | R: 5’-CTGGTGAGGCTGTCCGACTGTG-3’ |
| Collagen II | F: 5’- GTCCTACAATGTCAGGGCCA-3’ |
|  | R: 5’- ACCCCTCTCTCCCTTGTCAC-3’ |
| Aggrecan | F: 5’-CCGCTGGTCTGATGGACACT-3’ |
|  | R: 5’-AGGTGTTGGGGTCTGTGCAA-3’ |
| Collagen Ⅹ | F: 5’-TTCTGCTGCTAGTGTCCTTGACG-3’ |
|  | R: 5’-GGGATGAAGTATTGTGTCCTGGG-3’ |
| MMP-3 | F: 5’-GTACCAACCTATTCCTGGTTGC-3’ |
|  | R: 5’-CCAGAGAGTTAGATTTGGTGGG-3’ |
| IL-1 | F: 5’-CTCCAGCCACACTCCAACAGA-3’ |
|  | R: 5’-CACCCTAACACAAAACACGAT-3’ |
| IL-6 | F: 5'-AGAGGAGAC TTCACAGAGGA-3' |
|  | R: 5'-CCAGTTTGGTAG CATCCATC-3' |
| TNF-α | F: 5’-CCTACCTTCAGACCTTTCCAGAT-3’ |
|  | R: 5’-GGCCTTCCAAATAAATACATTCA-3’ |
| HIF-1α (FISH) | 5'-CAUCUGUUAGCACCAUAACAAAGCCAUCCA-3' |
| SDF-1 (FISH) | 5'-AGAUGUUUGACGUUGGCUCUGGCGACAUGG-3' |
